# Accuracy of the AI-Based Smart Scope® Test as a Point-of-Care Screening and Triage Tool Compared to Colposcopy: A Pilot Study

**DOI:** 10.7759/cureus.81212

**Published:** 2025-03-26

**Authors:** Manju A Talathi, Suchita Dabhadkar, Prakash P Doke, Varsha Singh

**Affiliations:** 1 Obstetrics and Gynecology, Bharati Vidyapeeth (Deemed to be University) Medical College, Pune, IND; 2 Community Medicine, Bharati Vidyapeeth (Deemed to be University) Medical College, Pune, IND; 3 Clinical Research, Periwinkle Technologies Pvt. Ltd., Pune, IND

**Keywords:** artificial intelligence (ai), cervical cancer, colposcopy, digital visual examination with acetic acid, point-of-care-test, smart scope®, via vili

## Abstract

Objectives

The primary objective of this study was to compare the screening accuracy of AI assessment with colposcopy. Secondary objectives included comparing the triaging accuracy of AI and colposcopy assessments against histopathology.

Methodology

This prospective, single-arm screening test assessment study was conducted at the obstetrics and gynecology department of Bharati Vidyapeeth (Deemed to be University) Medical College in Pune, India. The study included sexually active, nonpregnant women aged 25-65 years visiting the OPD for per-speculum examination. Women with a clinically unhealthy cervix detected during the examination were counseled, and those who provided consent were enrolled. Patients with a history of prior cervical cancer treatment or hysterectomy were excluded. A total of 130 women were enrolled. Each participant underwent colposcopy, Smart Scope^®^-AI (SS-AI) assisted visual inspection with acetic acid (VIA), and visual inspection with Lugol’s iodine during the same visit. Positive findings from any test led to a biopsy, with samples sent for histopathological analysis.

Results

Of the 130 women enrolled, 30 were referred for biopsy. Histopathology results were obtained for 18 consenting women. Using colposcopy as the reference standard (N = 130), the accuracy of SS-AI was 76.53%. When compared to histopathology (N = 18) as the gold standard, the accuracy of colposcopy and SS-AI was 63.67% and 83.33%, respectively. The sensitivity and specificity of SS-AI were both 83.33%, while colposcopy had a sensitivity of 83.33% and a specificity of 50%. Likelihood ratios for SS-AI were superior to those of colposcopy. These findings suggest that the SS-AI-assisted test, a digital VIA test, accurately detects positive and negative cervical lesions.

Conclusions

The SS-AI system demonstrated comparable effectiveness to colposcopy and has the potential to be used as a point-of-care screening and triaging tool in primary healthcare centers lacking colposcopy equipment for triaging purposes.

## Introduction

According to the 2022 report, cervical cancer ranks as the fourth most common cancer among women worldwide, with 662,301 new cases and approximately 348,874 deaths annually [1]. In India, it is the second most common cancer among women, contributing to 125,600 new cases and 77,947 deaths among women aged 20-84 years, as reported by WHO in 2022 [2]. The failure of cervical cancer screening programs in India is largely due to inadequate access to screening and healthcare services. The Pap smear test, a multi-visit screening method, is known for its low sensitivity [3-5].

Sankaranarayanan et al. have recommended visual inspection with acetic acid (VIA) and Lugol's iodine (VILI) as effective screening methods for preventing cervical cancer in low-resource settings, provided there is adequate training and ongoing quality assurance [6]. While WHO has endorsed the HPV DNA test as the primary screening method for the screen-and-treat approach [7], colposcopy evaluation and guided biopsy continue to be essential for triaging and diagnosis. However, colposcopy is not available in most community settings and requires highly skilled personnel, limiting its use to tertiary facilities.

To our knowledge, there are no reports of national cervical pre-cancer incidence rates. However, among women from various regions of India attending hospital OPDs, abnormal colposcopy findings on clinically unhealthy cervixes have been reported to range from 16% to 42% [8]. In Maharashtra, approximately 41% of colposcopy results are abnormal.

Implementing a point-of-care test capable of both screening and triaging is crucial to reduce dependency on colposcopy-based triaging and ensure the success of the screen-and-treat strategy. The Smart Scope^®^ (SS) test is a standard visual examination where cervix images are captured using the transvaginal digital device SS, and these digital images are automatically assessed through cloud-based AI to categorize lesions.

This study aimed to evaluate the feasibility of using the SS assisted by AI as a screening and triaging tool. The primary objective was to compare the screening accuracy of AI assessment with colposcopy. Additionally, to determine triaging accuracy, we planned to (a) compare AI assessment accuracy with histopathology and (b) compare colposcopy accuracy with histopathology.

## Materials and methods

Study design

This prospective, single-arm study involved a qualified and trained colposcopist performing colposcopy assessments, while a trained nurse conducted the index test.

Study period

The study was conducted at the obstetrics and gynecology department of Bharati Vidyapeeth (Deemed to be University) Medical College in Pune, India, from April 2022 to July 31, 2023, spanning 15 months.

Inclusion criteria

Sexually active women aged 25-65 years who visited the hospital’s OPD presenting with symptoms such as white discharge, vaginal pruritus, lower abdominal pain, abnormal per vaginal bleeding, or a clinically unhealthy cervix detected during the per-speculum examination.

Exclusion criteria

Pregnant women and those with a history of prior treatment for cervical cancer or hysterectomy were excluded from the study.

Devices

**Figure 1:**
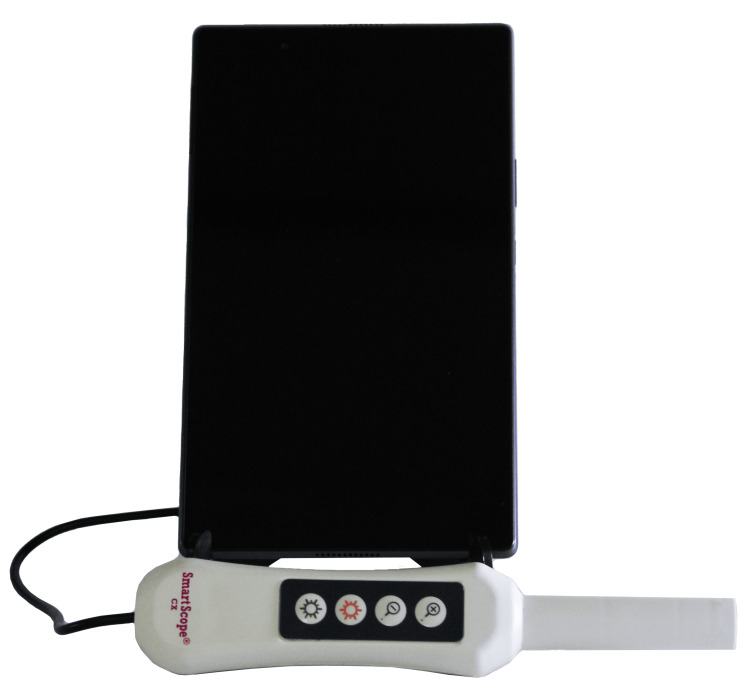
SS and accompanying tablet SS, Smart Scope^®^

The SS (model CX2.0) is a digital, portable, transvaginal device (Figure 1) with a focal length of 3-4 cm and 10X magnification. It is paired with a tablet equipped with Net4Medix^®^ software, which stores cervix images and related patient data. The SS^®^ test operates on the VIA-VILI principle, capturing cervix images using the SS and saving them on the tablet for further analysis.

The SS is connected to a tablet equipped with built-in software for data storage and AI-enabled image assessment. Cervical images are categorized into four color-coded groups based on the presence, absence, and severity of lesions. A green color indicates a normal cervix, amber signifies benign conditions such as infection, inflammation, or squamous metaplasia, brown (high risk amber) indicates likely low-grade lesions, and red indicates potential high-grade lesions or carcinoma.

For colposcopy, a BORZE digital colposcope was used, featuring a green filter, TV camera, CTV display, a focal length of 250 millimeters, magnification ranging from 7.5 to 10X, and a working distance of 25 cm. Colposcopy assessments were reported following the 2011 International Federation for Cervical Pathology and Colposcopy nomenclature.

Investigation

Nonpregnant women visiting the gynecology OPD underwent a naked-eye per-speculum examination. Women with suspicious or unhealthy cervixes were counseled, and those who provided consent were enrolled in the study. A total of 130 women underwent both colposcopy assessment and SS-AI-assisted standard VIA-VILI tests [9]. To avoid bias, SS-AI and colposcopy assessments were performed simultaneously in the same session by two different individuals.

During the examination, the cervix was cleaned with saline, followed by the sequential application of 5% acetic acid (AA) and Lugol's iodine (LI). Cervical images were captured using both the SS and colposcope at three stages: after saline cleaning, after AA application, and after LI application. The cloud-based AI model assessed the SS images, while colposcope images were independently evaluated by an expert colposcopist.

Women classified as high-risk amber (HRA) or red on AI were considered positive and scheduled for biopsy. In colposcopy, women with minor or worse lesions were classified as positive; those with major or worse lesions were recommended for biopsy, while biopsy was optional for those with minor lesions. Symptomatic women who tested negative on both tests were offered conservative surgical management, such as a loop electrosurgical excision procedure, with tissue samples sent for histopathology.

Sample size determination

The study aimed to evaluate whether the SS test with AI could replace colposcopy for triaging. Women presenting at the hospital OPD with gynecological complaints and clinically unhealthy cervixes were enrolled. Based on the 41% abnormal colposcopy rate reported in the OPD setting in Maharashtra [8], with an expected sensitivity of 90% and specificity of 75%, the estimated sample size was calculated to be 125 women.

Statistical analysis

Histopathology at the cervical intraepithelial neoplasia I (CIN I)+ cutoff was considered the gold standard. In the absence of histopathology data, colposcopy was used as the reference standard. Screen positives were defined as suspected low-grade lesions or higher on colposcopy and as HRA or worse on AI assessment.

Statistical analysis was performed using IBM SPSS Statistics for Windows, Version 20.0 (Released 2011; IBM Corp., Armonk, NY, USA). For quantitative (continuous) data, arithmetic means ± SD were calculated, while frequencies (%) were used for categorical data. P-values of <0.05 were considered statistically significant. The chi-square test was applied to evaluate the performance of SS-AI against histopathology and colposcopy. Performance metrics such as positive likelihood ratio, negative likelihood ratio, area under the curve (AUC), accuracy, sensitivity, and specificity were considered to determine the effectiveness of the test.

Ethics statement

The study received approval from the Institutional Ethics Committee (IEC/2022/17). Informed written consent was obtained from all participants after proper counseling.

Registration

The study was registered in the Clinical Trials Registry - India (CTRI) (CTRI/2022/04/041568).

## Results

**Figure 2:**
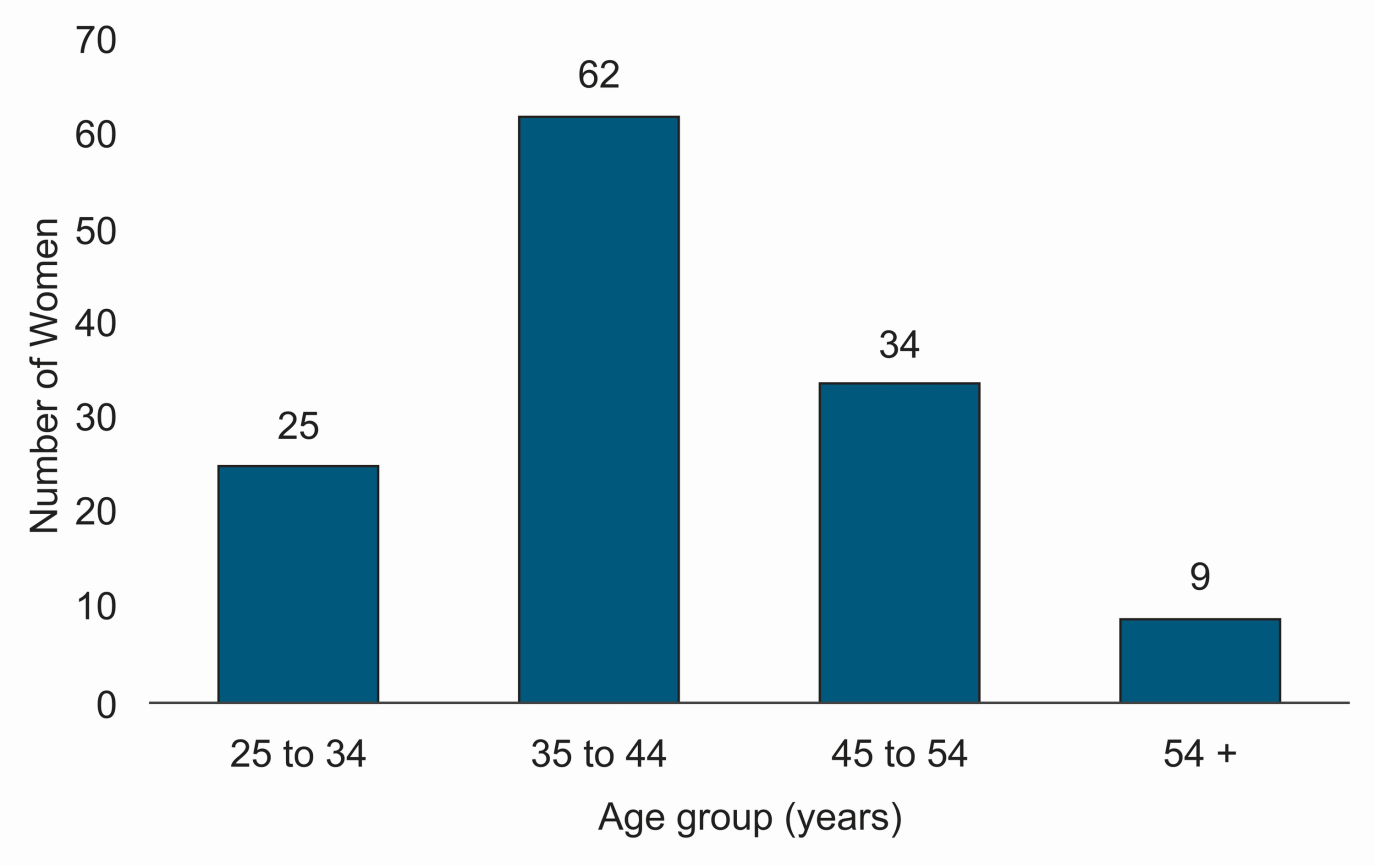
Age distribution of enrolled women (N = 130)

A total of 130 women were enrolled in the study based on the inclusion and exclusion criteria. The majority of participants (48%) were from the late reproductive age group of 35-44 years, followed by 26% from the 45-54 years age group (Figure 2).

Frequency distribution of assessment outcome by SS-AI, colposcopy, and histology

Table 1 presents the frequency distribution of screening and diagnostic outcomes for the enrolled patients. All 130 women underwent assessment through both colposcopy and the digital VIA-VILI test using the SS.

**Table 1:** Frequency distribution of test results CIN I, cervical intraepithelial neoplasia I; HRA, high-risk amber; NILM, negative for intraepithelial lesion or malignancy

Test	Classification	Frequency	Percentage (%)
Colposcopy	NILM	29	22.3
	Benign	78	60.0
	Minor lesion	20	15.4
	Major lesion	3	2.3
	Total	130	100.0
AI	Green	68	52.3
	Amber	43	33.1
	HRA	14	10.8
	Red	5	3.8
	Total	130	100.0
Histopathology	Benign	12	66.7
	CIN I	6	33.3
	Total	18	100.0

**Figure 3:**
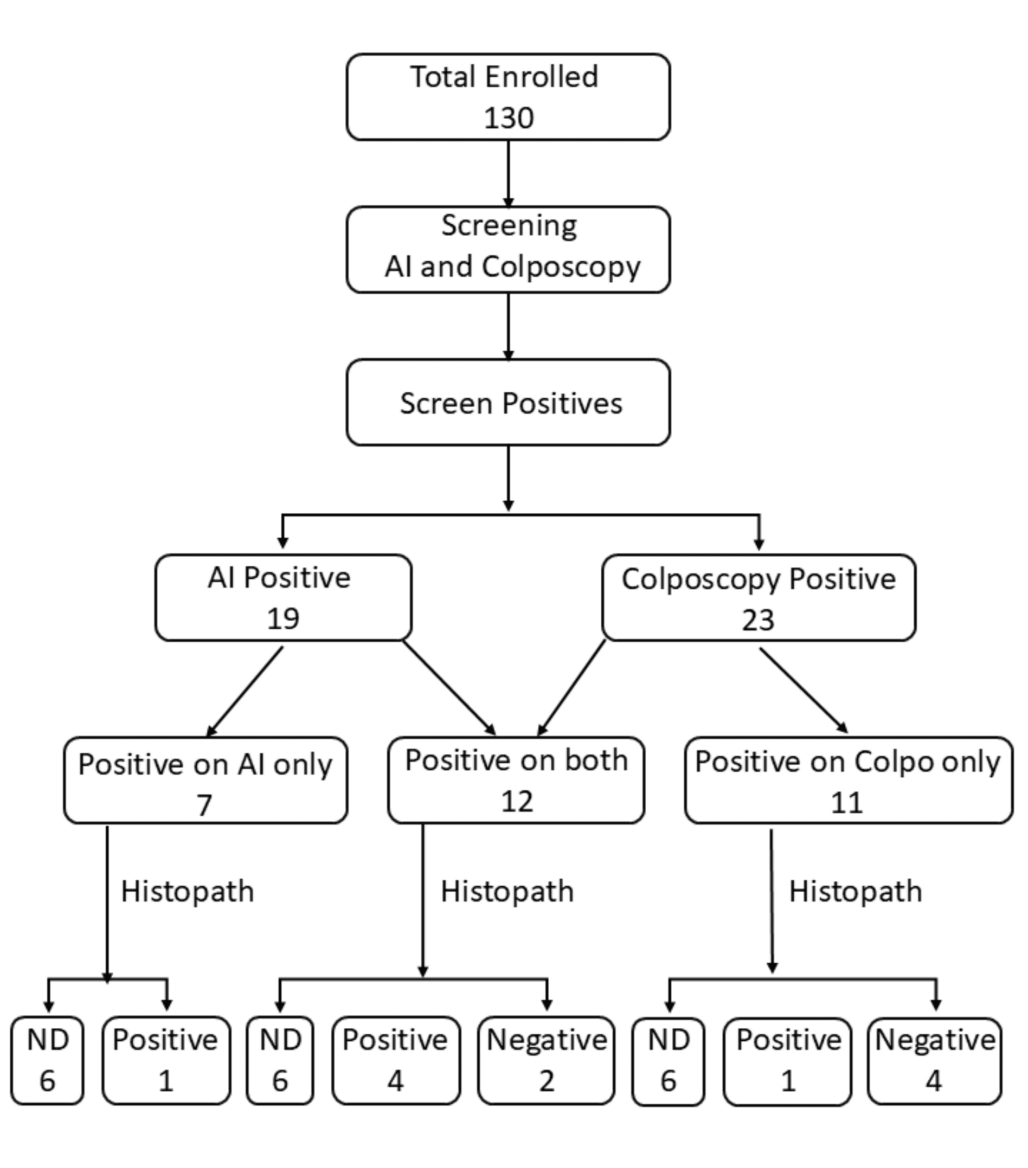
Distribution of screen-positive cases identified by colposcopy and SS-AI Histopath, histopathology; ND, histopathology not done; SS-AI, Smart Scope^®^ AI

Out of the 130 enrolled women, 107 were negative, while 23 (17.7%) were found to have minor grade or higher lesions on colposcopy (Figure 3). Among these 23 positive cases, five were confirmed as CIN I lesions through histopathology. When the same group of 130 women was screened using SS-AI, 111 tested negative, and 19 (14.6%) were identified as positive by SS-AI (Figure 3). Of these 19 positive cases, five were confirmed as CIN I lesions. Twelve women tested positive on both colposcopy and SS-AI, while 100 women were negative on both tests (Figure 3).

In addition, histopathology results were available for six symptomatic women who were screen-negative. This brought the total number of histopathology results to 18. Histopathology examination revealed that six women (33.3%) had CIN I lesions, while 12 women (66.7%) had benign lesions (Table 1). No cases of CIN II or carcinoma were reported. All six symptomatic women had histopathology results negative for pre-cancer or cancer.

Comparison of AI with colposcopy

AI assessment was compared with colposcopy assessment across all 130 cases (Table 2).

**Table 2:** Comparison of screen-positive and screen-negative results between SS-AI and colposcopy (N = 130) SS-AI, Smart Scope^®^ AI

SS-AI	Colposcopy		
	Negative	Positive	Total
Negative	100	11	111
Positive	7	12	19
Total	107	23	130

**Figure 4:**
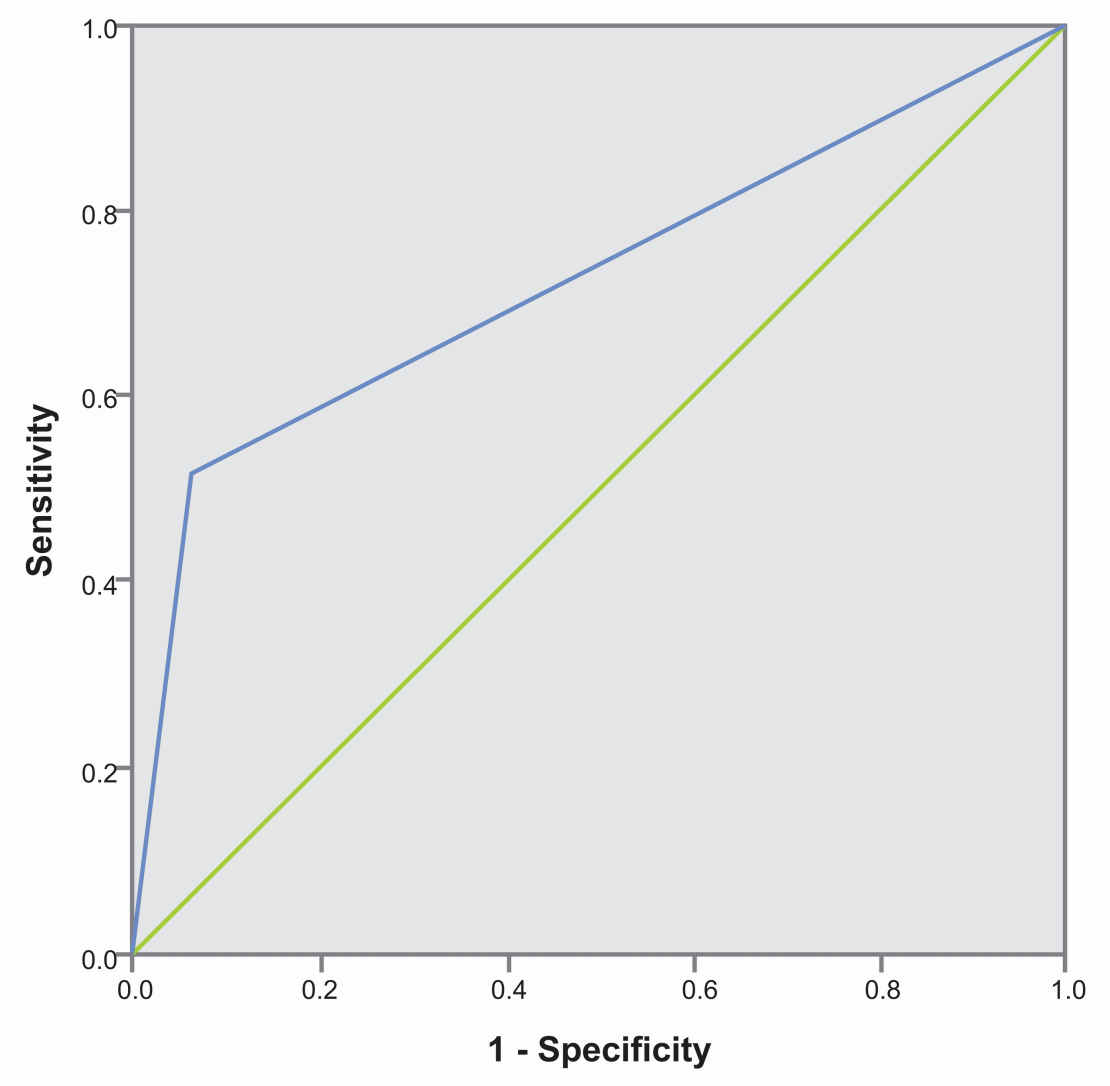
ROC curve comparing SS-AI with colposcopy The blue line represents SS-AI (with colposcopy minor grade+ as the reference test), while the green line indicates a random curve. ROC curve, receiver operating characteristic curve; SS-AI, Smart Scope^®^ AI

Statistical values, along with confidence intervals for SS-AI compared to colposcopy as the reference standard, are presented in Table 3. The results indicate that AI achieved a high specificity of 93.46%, with an AUC of 0.73 (95% CI: 0.6-0.86, p = 0.01) (Figure 4).

**Table 3:** Statistical performance of SS-AI compared to colposcopy as reference standard (N = 130) ^* ^The value depends on the prevalence (41%) of abnormal findings. SS-AI, Smart Scope^®^ AI

Statistics	Value	95% CI
Sensitivity	52.17%	30.59-73.18%
Specificity	93.46%	86.98-97.33%
Positive likelihood ratio	7.98	3.53-18.04
Negative likelihood ratio	0.51	0.33-0.79
Positive predictive value^*^	84.71%	71.02-92.61%
Negative predictive value^*^	73.77%	64.66-81.21%
Accuracy^*^	76.53%	68.30-83.52%

Comparison of colposcopy and AI with histopathology

**Figure 5:**
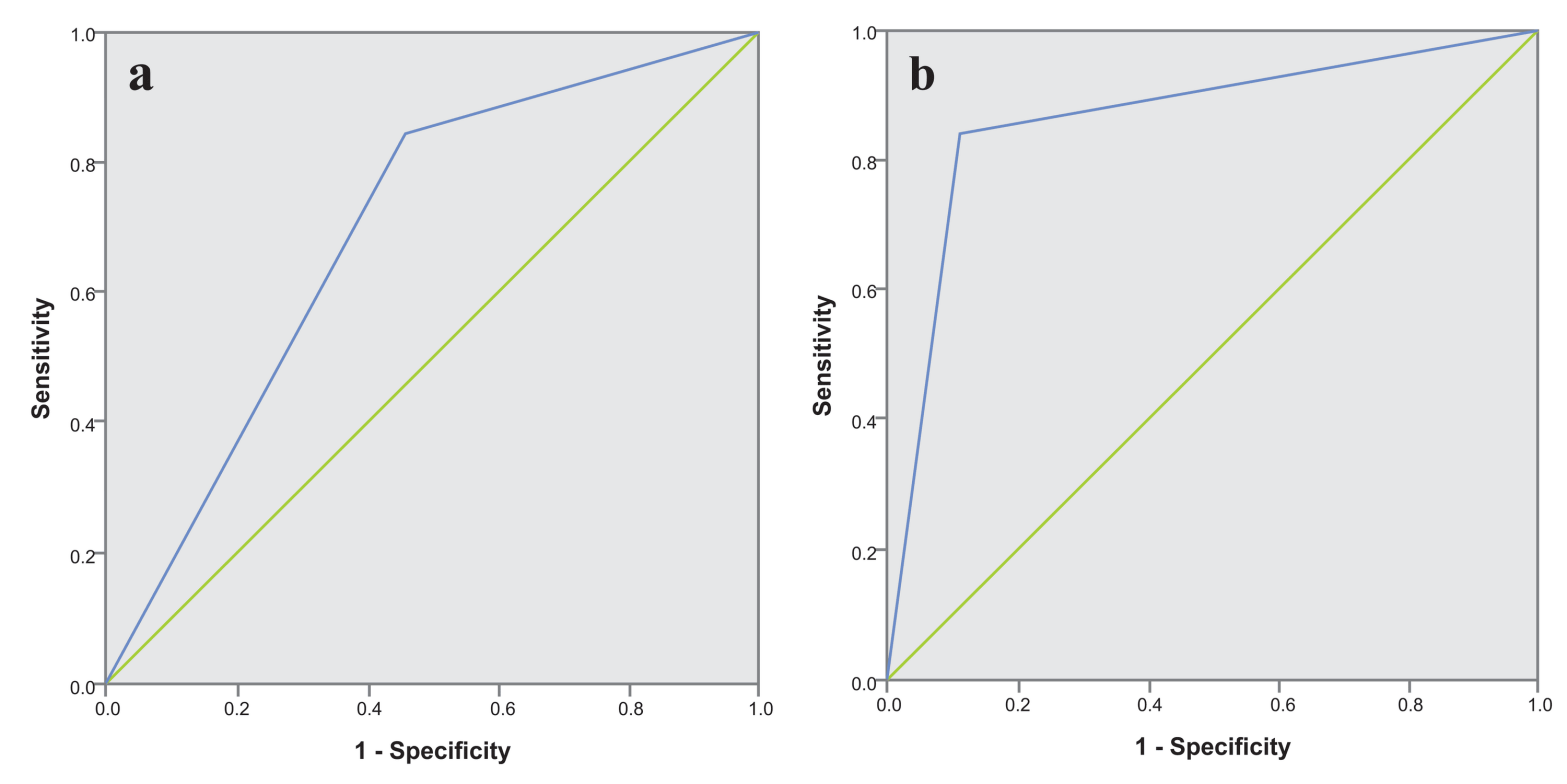
ROC curves comparing screening tests against histopathology: (a) the blue line represents colposcopy performance; (b) the blue line represents SS-AI performance The green line represents a random curve. ROC curve, receiver operating characteristic curve; SS-AI, Smart Scope^®^ AI

Among the 130 enrolled women, histopathology reports were available for 18 cases (Table 4). Of these, colposcopy identified five true positive cases and six true negative cases. The sensitivity and specificity of colposcopy were 83.33% and 50%, respectively (Table 5). The negative predictive value of colposcopy was 81.19%, with an overall accuracy of 63.67%. The positive likelihood ratio was 1.67, while the negative likelihood ratio was 0.33. The AUC for colposcopy was 0.67 (95% CI: 0.4-0.93, p = 0.134) (Figure 5a). Colposcopy missed one confirmed CIN I lesion and overestimated six benign lesions.

**Table 4:** Distribution of colposcopy and SS-AI assessments against histopathology (N = 18) SS-AI, Smart Scope^®^ AI

Histopathology	Colposcopy		SS-AI	
	Negative	Positive	Negative	Positive
Negative (12)	6	6	10	2
Positive (6)	1	5	1	5
Total (18)	7	11	11	7

**Table 5:** Statistical analysis outcome of screening tests against histopathology as gold standard (N = 18) ^*^ The value depends on the prevalence (41%) of abnormal findings. SS-AI, Smart Scope^®^ AI

Statistics	Colposcopy		SS-AI	
	Value	95% CI	Value	95% CI
Sensitivity	83.33%	35.88-99.58%	83.33%	35.88-99.58%
Specificity	50.00%	21.09-78.91%	83.33%	51.59-97.91%
Positive likelihood ratio	1.67	0.85-3.26	5	1.34-18.62
Negative likelihood ratio	0.33	0.05-2.18	0.2	0.03-1.22
Positive predictive value^*^	53.66%	37.22-69.35%	77.65%	48.27-92.83%
Negative predictive value^*^	81.19%	39.80-96.57%	87.80%	54.15-97.77%
Accuracy^*^	63.67%	38.13-84.56%	83.33%	58.58-96.42%

Similarly, AI assessments were compared with histopathology outcomes for the same 18 women (Table 4). AI accurately identified five positive cases and 10 negative cases, resulting in a sensitivity and specificity of 83.33% each (Table 5). The positive predictive value and negative predictive value were 77.65% and 87.8%, respectively, with an overall accuracy of 83.33%. The positive and negative likelihood ratios were 5 and 0.2, respectively. The AUC for AI was 0.833 (95% CI: 0.62-1.0, p = 0.025), as shown in Figure 5b. AI missed one CIN I case and overestimated two benign cases. Of the six CIN I cases confirmed by histopathology, AI and colposcopy each missed one case, although they were different cases.

## Discussion

To significantly reduce cervical cancer incidences, implementing a screen-and-treat policy is essential. This approach requires healthcare workers to make triaging decisions during screening, highlighting the need for efficient tools like AI to support decision-making at primary health centers.

Allahqoli et al. reviewed various machine-learning algorithms developed and tested for different gynecological issues [10]. AI has been applied to guide biopsies and to train junior colposcopists and gynecologists [11]. When inexperienced colposcopists used AI for image interpretation, improvements were observed in sensitivity, specificity [12,13], accuracy, and AUC [14]. AI models that incorporated predictors such as age, number of sexual partners, contraceptive use, education level, and presence of HPV genotypes achieved accuracy rates ranging from 70% to 100% [10]. Without AI, cytology screening accuracy for CIN I-III and AIS ranged from 67% to 98.27%. However, applying AI to cytology assessment increased accuracy to between 80% and 100%. For detecting CIN II+ lesions, AI-assisted colposcopy showed sensitivity ranging from 71.9% to 98.22% and specificity between 51.8% and 96.2% [10]. One study using SS reported an AI screening accuracy of 84.04% [15].

AI has also been tested as a co-test for colposcopy [16] and even as a potential replacement for conventional colposcopy [17]. In several studies, AI was developed for triaging purposes [17] and for aiding clinicians in selecting biopsy sites [11]. However, many of these reports are based on retrospective diagnostic studies [11,18]. For instance, a study involving the EVA-Visualcheck^™^ colposcope found that the AI algorithm did not improve diagnostic speed, with congruence rates of 54.2% with histopathology and 47.9% with trained colposcopists [19].

Conversely, a Korean study using the Cerviary AI^®^ system reported improved sensitivity compared to experienced colposcopist interpretations [20]. Similar findings were noted by Ito et al. when AI was applied to 463 colposcopy images, enhancing diagnostic accuracy for CIN II-III and invasive cancer [21]. Asiedu et al. reported an AI sensitivity of 81% and specificity of 78% when paired with the digital Pocket Colposcope [17]. Shamsunder et al. reported 90.3% sensitivity and 75.3% specificity when AI was used with the SS device [15].

The findings of the current study align with these positive outcomes [15,17,20,21]. Accuracy was used as the efficiency parameter, and the results demonstrated that the accuracy of SS-AI was considerably higher than that of colposcopy when histopathology was considered the gold standard. While the sensitivity of SS-AI was comparable to colposcopy, its specificity and likelihood ratios were superior.

A notable observation was that colposcopists, particularly in regions with low screening rates, tend to overestimate screen positives due to concerns about losing follow-up cases. This tendency results in lower specificity and accuracy of colposcopy when compared to histopathology. However, when colposcopy was used as the reference standard, SS-AI once again demonstrated high accuracy and a positive likelihood ratio. The relatively low sensitivity of SS-AI could be attributed to the overestimation by colposcopy.

These statistical findings suggest that SS-AI is not inferior to colposcopy, offering a viable alternative for cervical cancer screening.

Strengths and limitations

The SS-AI test was conducted by a minimally trained nurse using the portable SS device enabled with AI, highlighting its potential for use by healthcare workers with limited training. The nurse and colposcopist conducted their assessments independently, eliminating potential bias. Additionally, the SS-AI test is supported by software that allows for remote triaging, making it suitable for resource-limited settings. However, the study’s conclusions are limited by the small number of histopathology datasets, as many participants were unwilling to undergo a biopsy. Moreover, since this was a pilot study conducted at a single center, the lack of diversity in the sample size limits the generalizability of the findings.

## Conclusions

A point-of-care digital VIA-VILI-based AI-assisted SS test was implemented for immediate screening and triaging. When comparing SS-AI to colposcopy as a reference test, SS-AI demonstrated a high specificity of 93.46% and an accuracy of 76.53%, though its sensitivity was relatively low at 52.17%. This lower sensitivity is likely due to the use of colposcopy, a subjective test with a high false positive rate, as the reference standard. When the performance of SS-AI and colposcopy was further evaluated using histopathology as the gold standard, both methods showed an equal sensitivity of 83.33%. However, SS-AI achieved better specificity (83.33%) compared to colposcopy (50%). Additionally, the positive likelihood ratio of SS-AI was 5, significantly higher than that of colposcopy (1.67), while the negative likelihood ratios were 0.2 for SS-AI and 0.33 for colposcopy. These results suggest that the AI-assisted SS test is as effective as colposcopy and could serve as a reliable point-of-care screening and triaging tool in primary healthcare settings where colposcopy is unavailable.
